# Supplementary material for: Expression Profiling Identified TRPM7 and HER2 as Potential Targets for the Combined Treatment of Cancer Cells
Source: Cells. 2024 Oct 31;13(21):1801. doi: 10.3390/cells13211801 (PMC11545334; doi:10.3390/cells13211801)

# Suppl. Figure S1

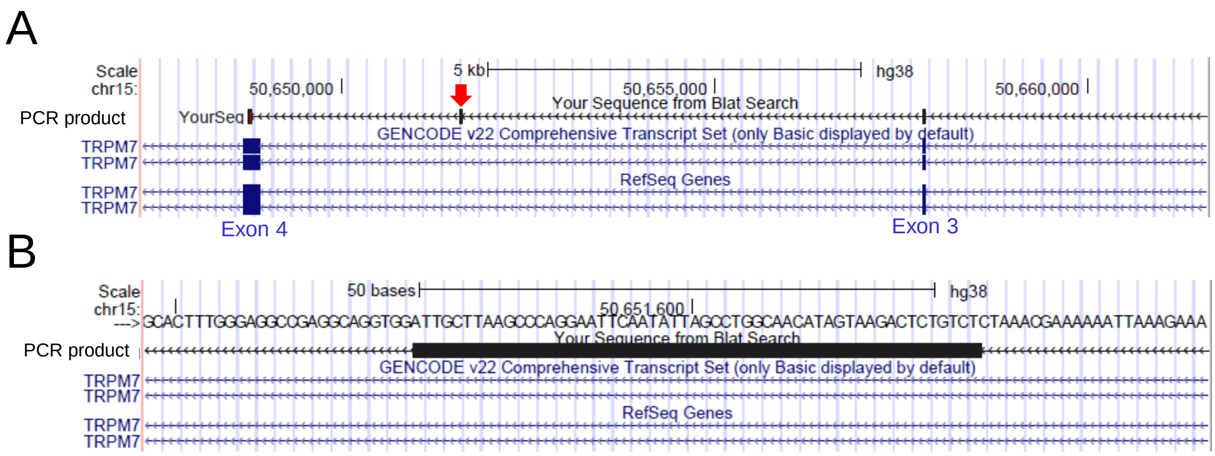


C

TGAGCACTTTGACCAAGAGGGATGTGTATATATTATACCAAGTTCCAAGGACCCTCACAGAT
GCCTTCCAGGATGTCAAATTTGTCAGCAACTCGTCAGAGACAGAGTCTTACTATGTTGCCAG
GCTAATATTGAATTCCTGGGCTTAAGCAATCAGGCAATAGAAGAATGGTATGTGGAAAAGCA
TACAGAACAGAG

Suppl. Figure S2

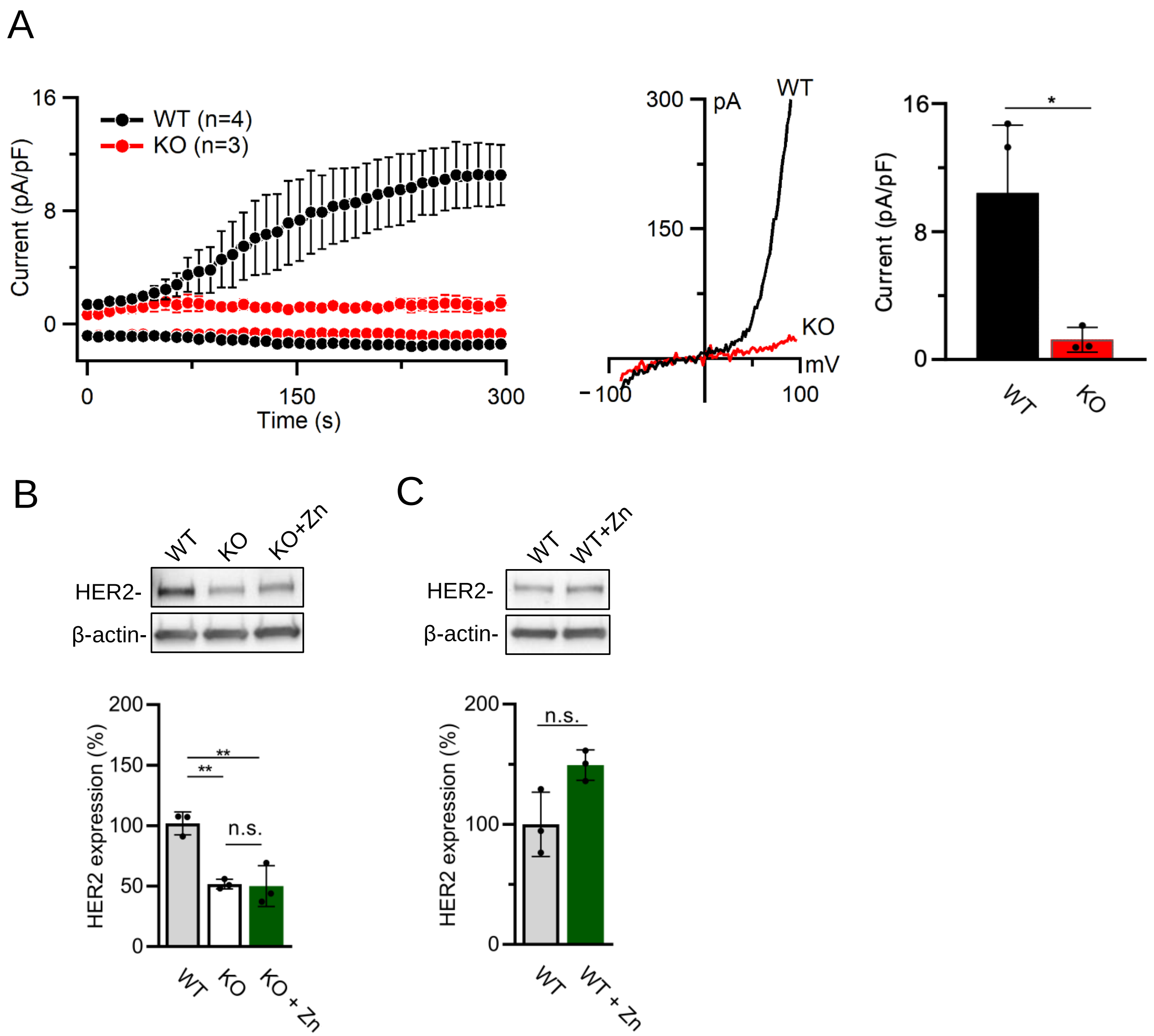

# Suppl. Figure S3

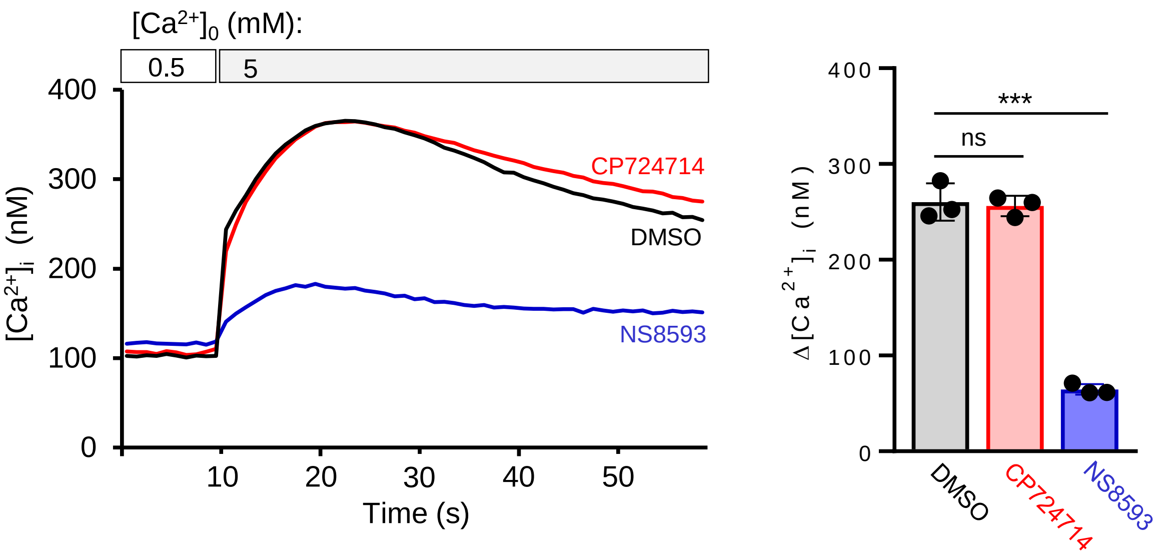

Suppl. Figure S4

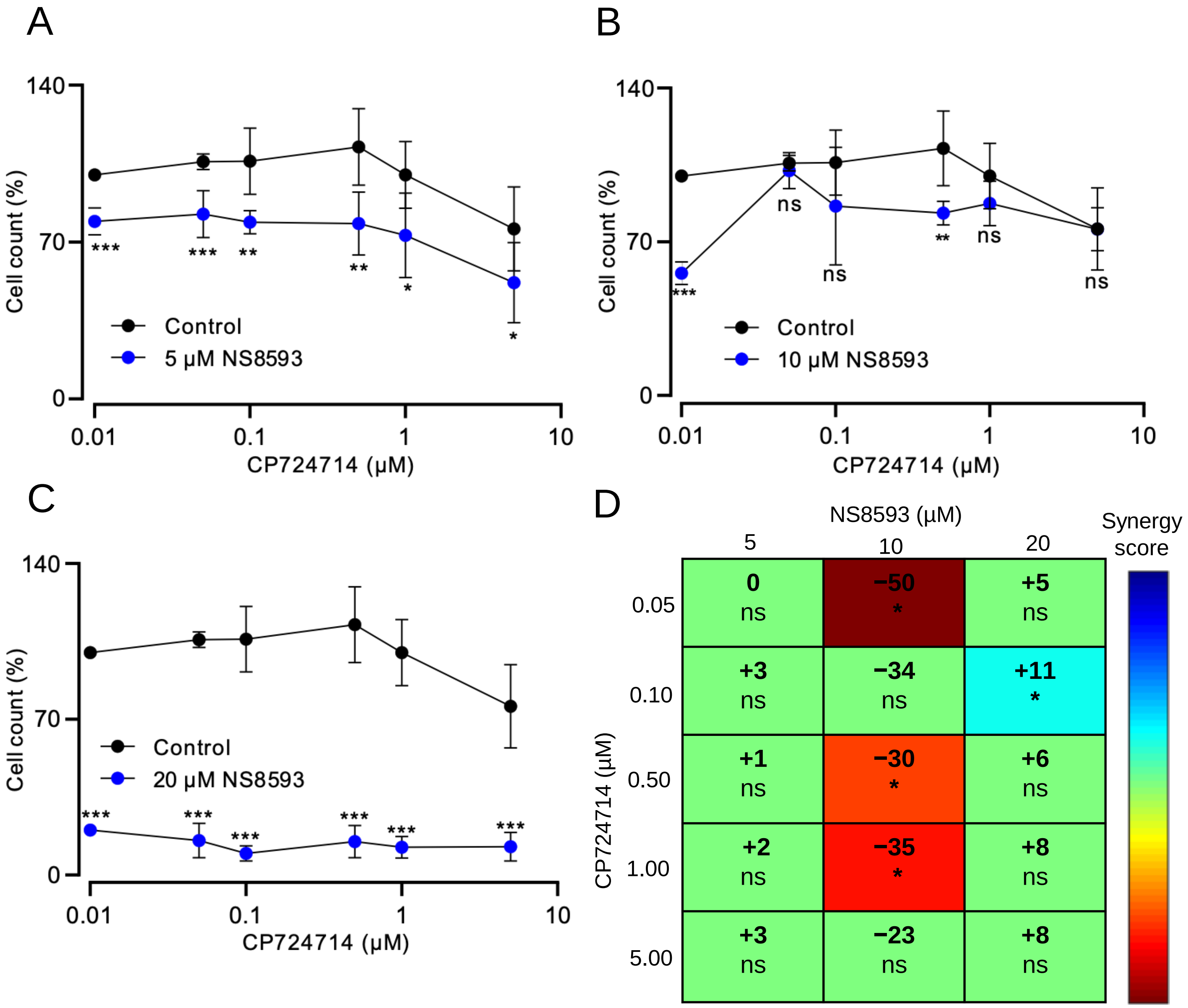

Supplement: Supplementary file 1 [file cells-13-01801-s001.zip › Suppl Figures.pdf]
