# Supplementary material for: Expression Profiling Identified TRPM7 and HER2 as Potential Targets for the Combined Treatment of Cancer Cells
Source: Cells. 2024 Oct 31;13(21):1801. doi: 10.3390/cells13211801 (PMC11545334; doi:10.3390/cells13211801)
Supplement: Supplementary file 1 [file cells-13-01801-s001.zip › Suppl Table S3.pdf]

Suppl. Table S3. Primers used for qRT-PCR assessment of HAP1 cells.

Primer	Sequence
ALPK1 for	5'-AGTGTGTTTCAGAGAGGCTGT-3'
ALPK1 rev	5'-GGGTGATCAATTACCCAAAGTTTC-3'
HER2 for	5'-AACTGCACCCACTCCTGTG-3'
HER2 rev	5'-AACCACACGCAGAGATGATGG-3'
HER3 for	5'-CTGATCACCGGCCTCAAT-3'
HER3 rev	5'-GGAAGACATTGAGCTTCTCTGG-3'
